# Supplementary material for: Deciphering the functional diversity of DNA-binding transcription factors in Bacteria and Archaea organisms
Source: PLoS One. 2020 Aug 21;15(8):e0237135. doi: 10.1371/journal.pone.0237135 (PMC7446807; doi:10.1371/journal.pone.0237135)
Supplement: S1 Fig — The abundance of TFs for each genome is shown on the y axis. Each dot corresponds to one genome. The model function (black line) and R2 are indicated for power: A) Alpha proteobacteria; B) Beta proteobacteria; C) Gamma proteobacteria; D) Cyanobacteria, and E) CFB (Cytophaga, Fusobacterium, and Bacteroides) group; and linear: F) Actinobacteria; G) Delta proteobacteria; H) Euryarchaeota; and I) Firmicutes. (PDF) [file pone.0237135.s001.pdf]

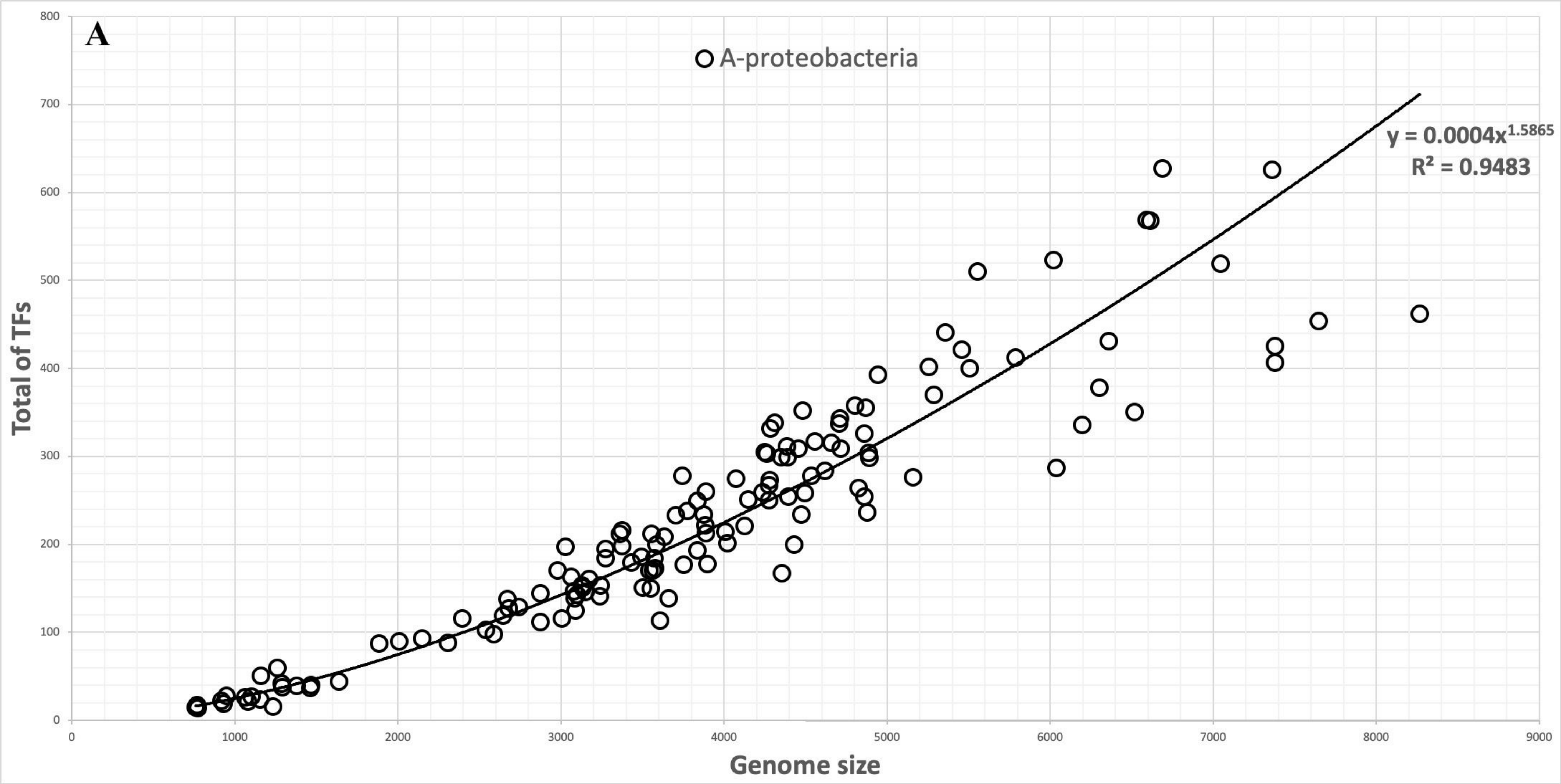
A
A-proteobacteria
y = 0.0004x^1.5865
R² = 0.9483
Total of TFs
Genome size
0
100
200
300
400
500
600
700
800
0
1000
2000
3000
4000
5000
6000
7000
8000
9000

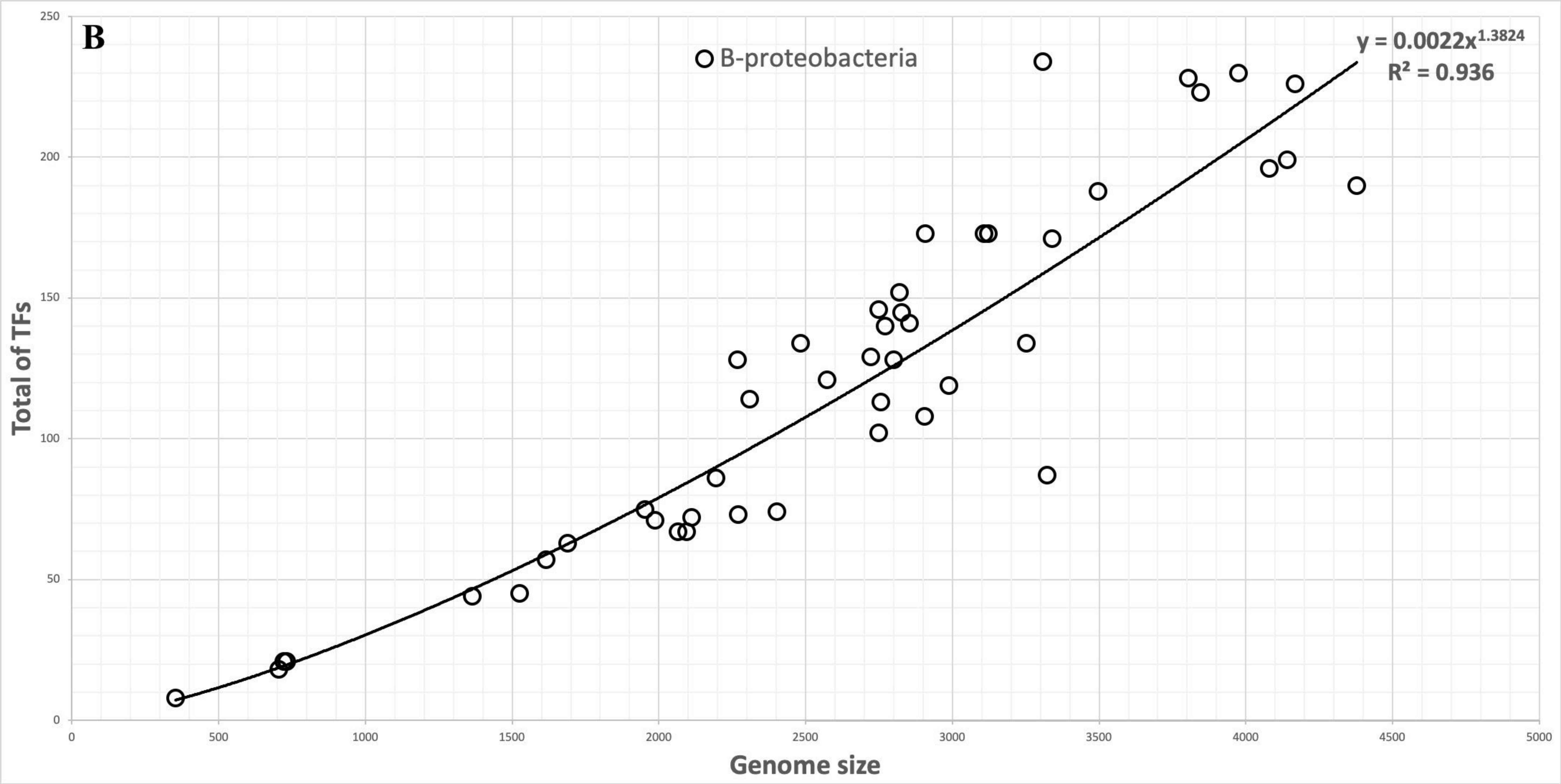
B
B-proteobacteria
y = 0.0022x^1.3824
R² = 0.936
Total of TFs
Genome size
0
50
100
150
200
250
0
500
1000
1500
2000
2500
3000
3500
4000
4500
5000

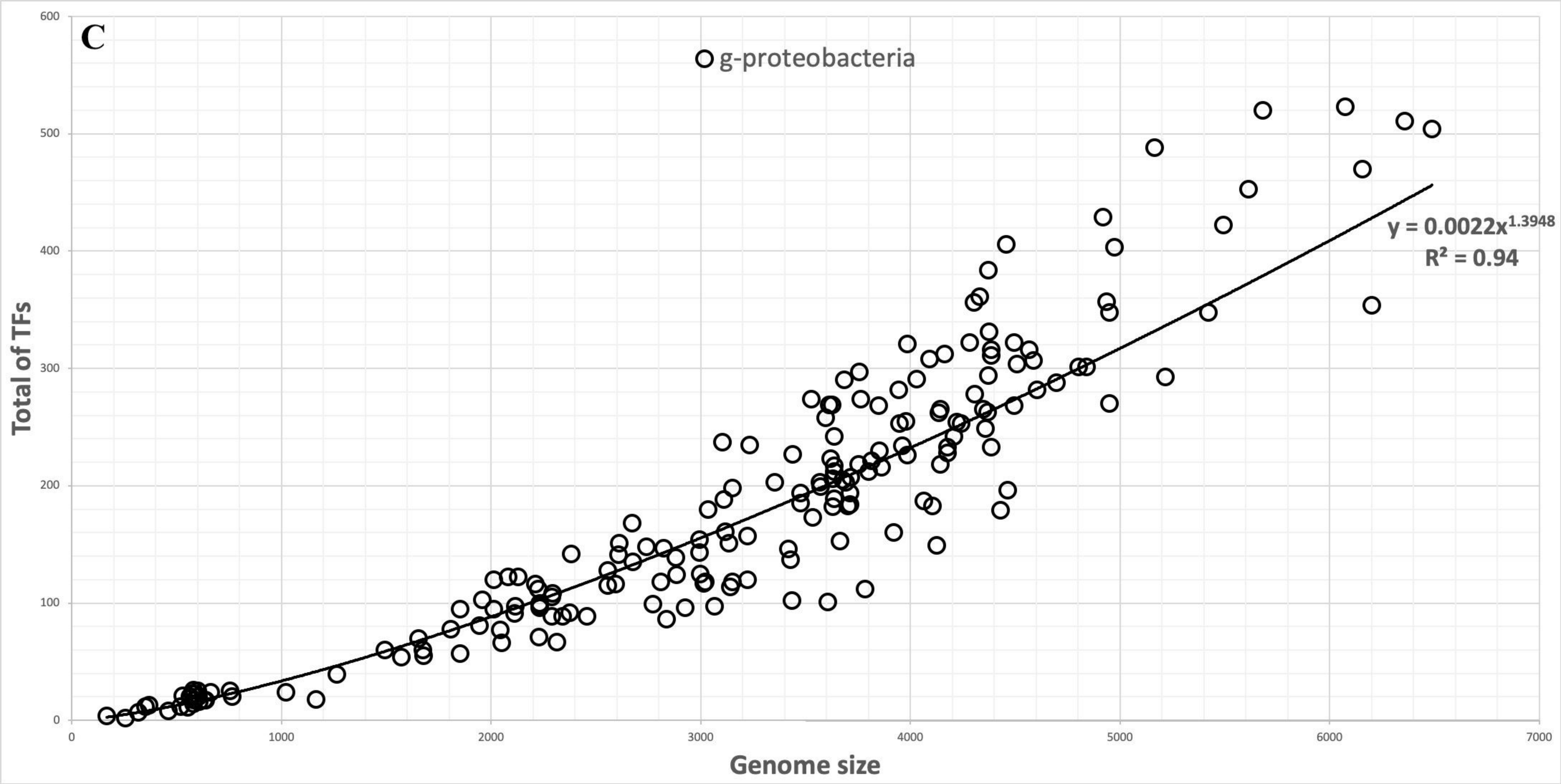
C
g-proteobacteria
y = 0.0022x^1.3948
R² = 0.94
Total of TFs
Genome size
0
100
200
300
400
500
600
0
1000
2000
3000
4000
5000
6000
7000

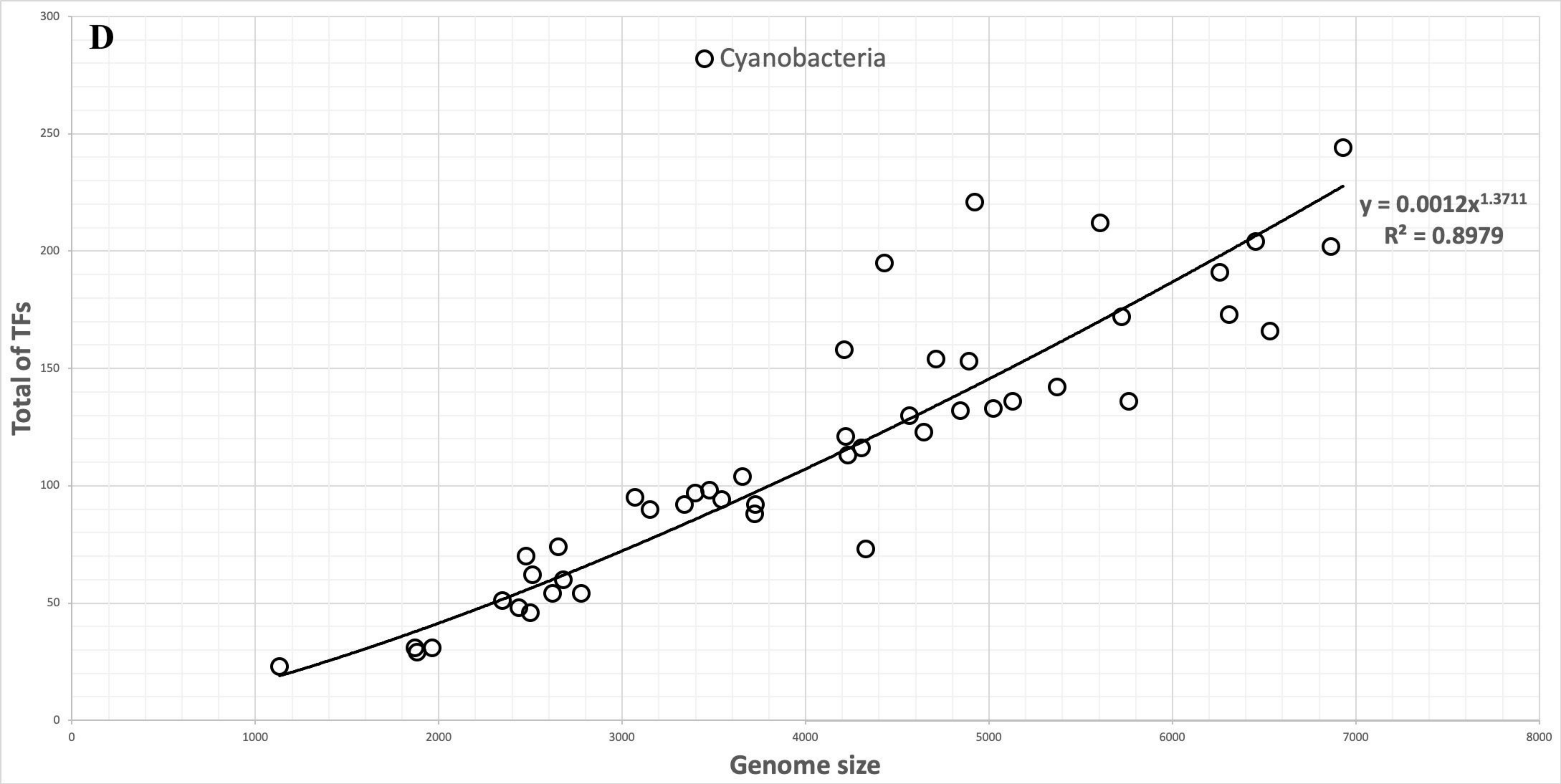
D
Cyanobacteria
y = 0.0012x^1.3711
R² = 0.8979
Total of TFs
Genome size
0
50
100
150
200
250
300
0
1000
2000
3000
4000
5000
6000
7000
8000

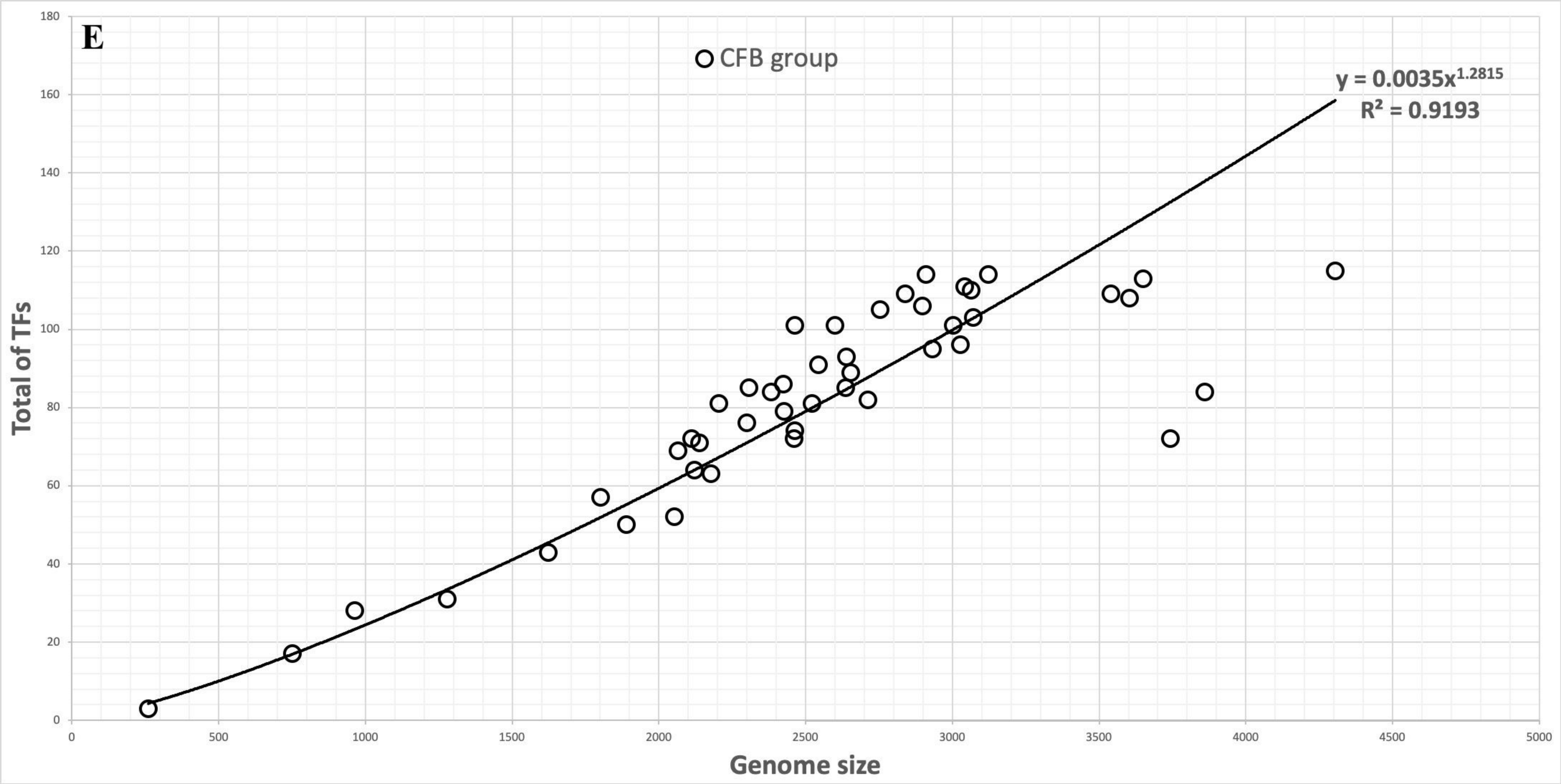

E
CFB group
y = 0.0035x^1.2815
R² = 0.9193
Total of TFs
Genome size
0
20
40
60
80
100
120
140
160
180
0
500
1000
1500
2000
2500
3000
3500
4000
4500
5000

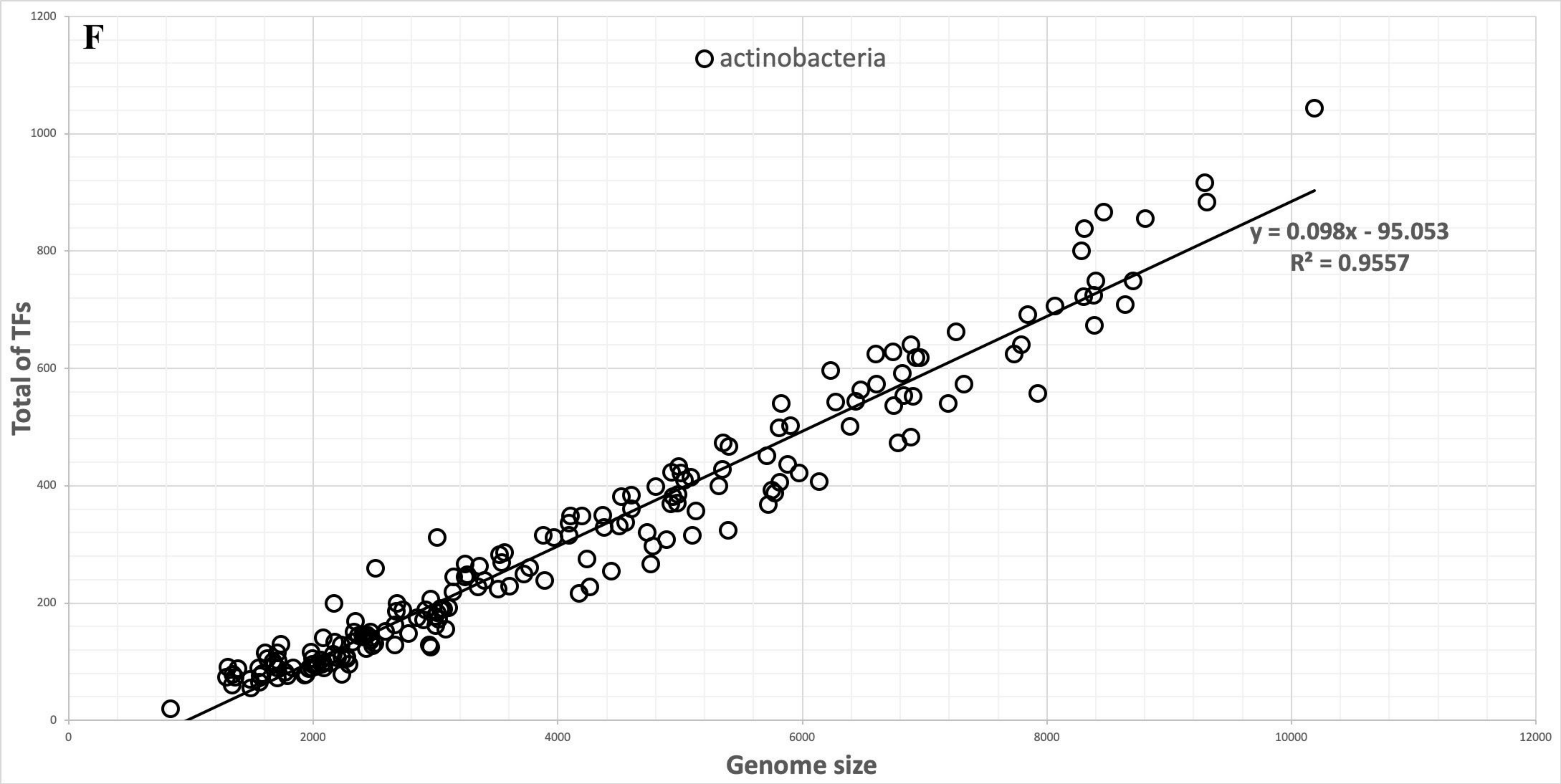

F
actinobacteria
y = 0.098x - 95.053
R² = 0.9557
Total of TFs
Genome size
0
200
400
600
800
1000
1200
2000
4000
6000
8000
10000
12000

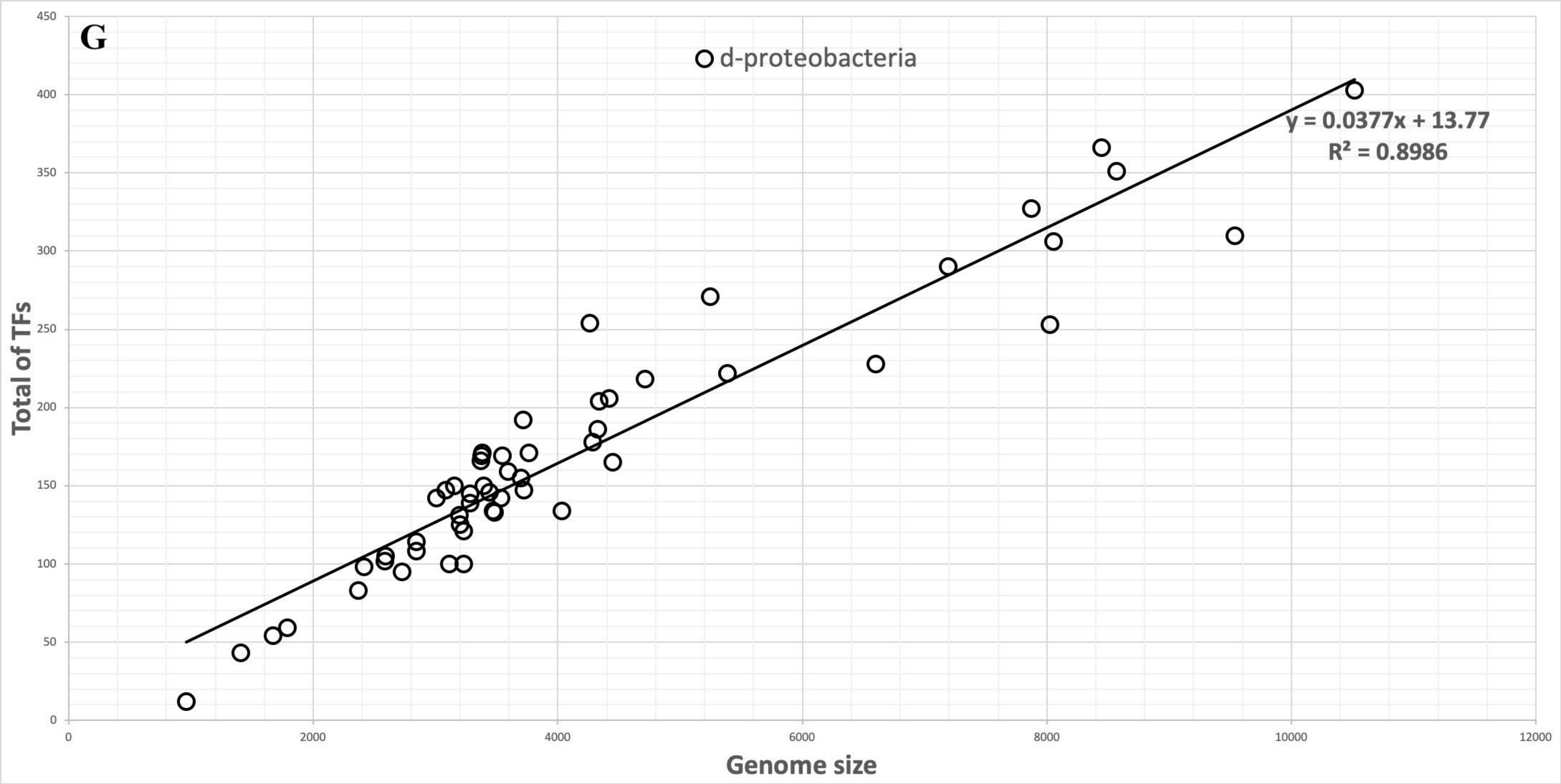

G
d-proteobacteria
y = 0.0377x + 13.77
R² = 0.8986
Total of TFs
Genome size
0
50
100
150
200
250
300
350
400
450
0
2000
4000
6000
8000
10000
12000

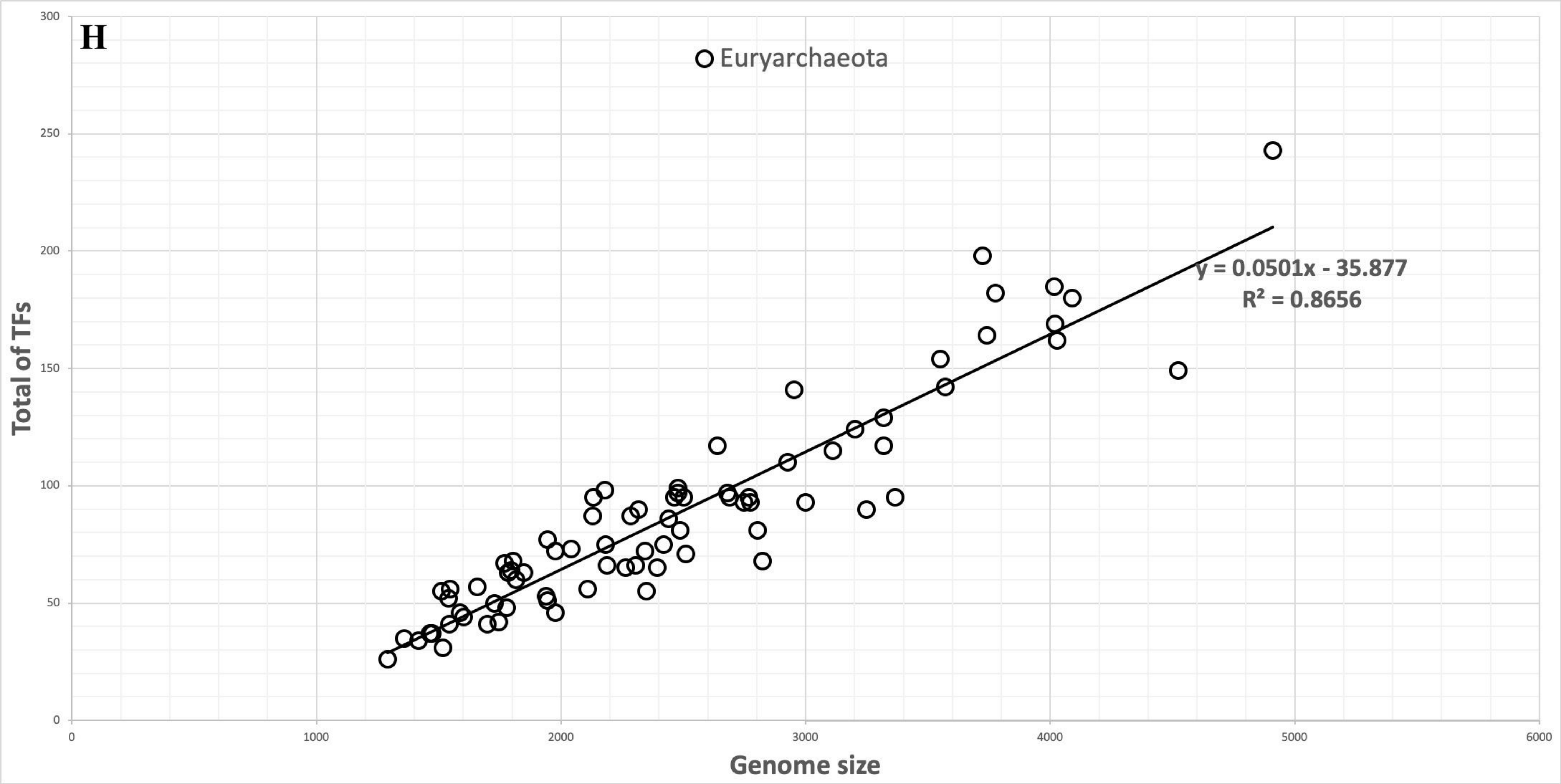
H
Euryarchaeota
y = 0.0501x - 35.877
R² = 0.8656
Total of TFs
Genome size
0
50
100
150
200
250
300
0
1000
2000
3000
4000
5000
6000

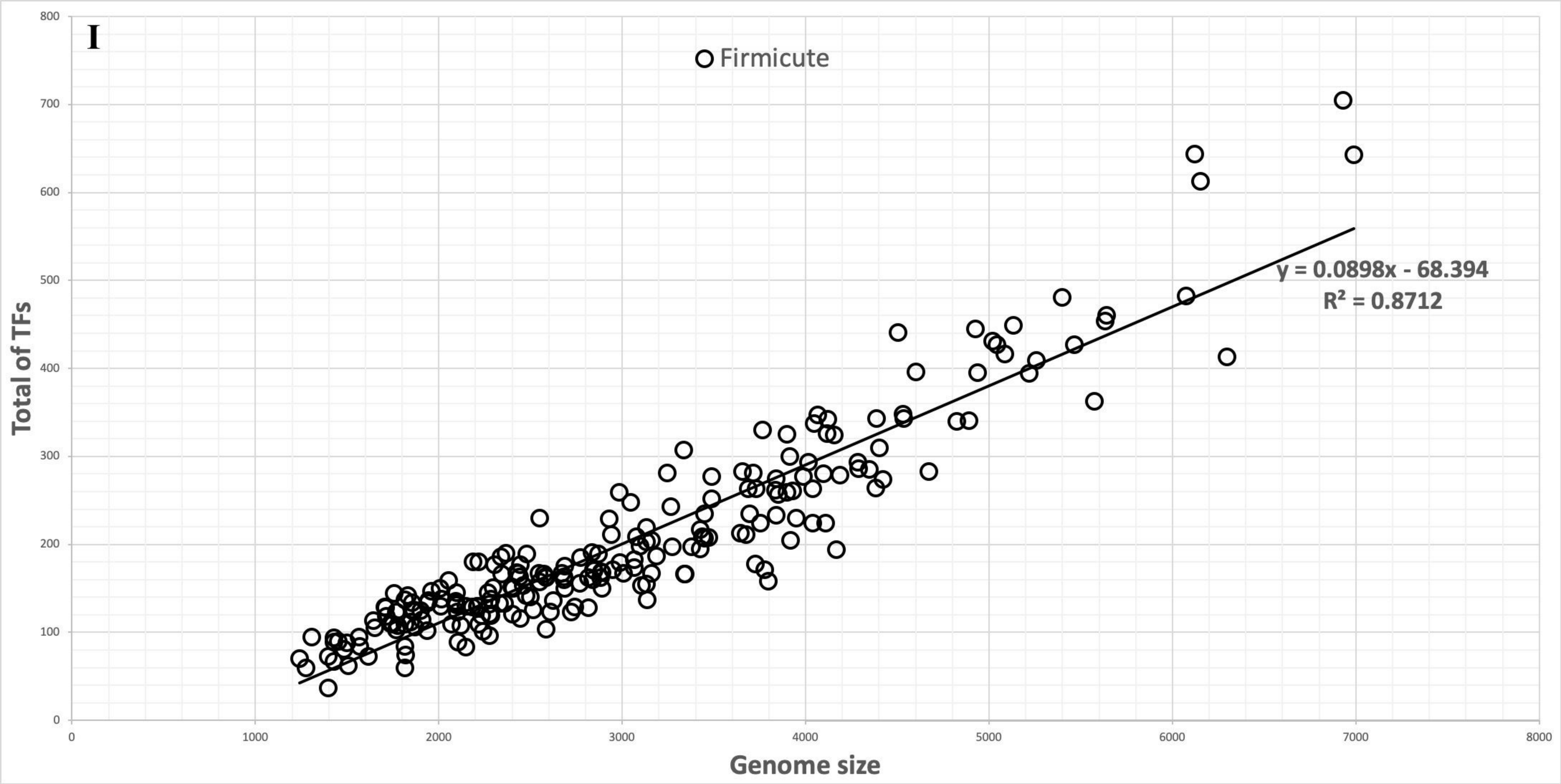
I
Firmicute
y = 0.0898x - 68.394
R² = 0.8712
Total of TFs
Genome size
0
100
200
300
400
500
600
700
800
0
1000
2000
3000
4000
5000
6000
7000
8000
